# Grafting Causes Physiological Changes and Promotes Adventitious Root Formation in Rejuvenated Soft Shoots of *Taxodium hybrid* ‘Zhongshanshan’

**DOI:** 10.3390/plants12010201

**Published:** 2023-01-03

**Authors:** Zhiquan Wang, Qin Shi, Peipei Chen, Feng Sun, David Creech, Zhiguo Lu, Yunlong Yin, Chaoguang Yu

**Affiliations:** 1 Institute of Botany, Jiangsu Province and Chinese Academy of Sciences, Nanjing 210014, China; 2 Jiangsu Key Laboratory for the Research and Utilization of Plant Resources, Nanjing 210014, China; 3 Jingjiang Greening Engineering Co., Ltd., Jingjiang 214500, China; 4 College of Forestry and Agriculture, Stephen F. Austin State University, Nacogdoches, TX 75962, USA

**Keywords:** *Taxodium hybrid* ‘Zhongshanshan’, rejuvenation, cutting, physiological changes

## Abstract

*Taxodium hybrid* ‘Zhongshanshan’ has been widely used as a timber tree in river network areas and coastal regions and is mainly propagated by cuttings. However, when trees age, their capacity to form adventitious roots becomes weaker. We successfully enhanced the rooting ability of shoots in *T. hybrid* ‘Zhongshanshan 302’ by their rejuvenation based on grafting. We recorded temporal variation in endogenous auxin, abscisic acid (ABA), gibberellins (GAs), trans-zeatin-riboside (TZR), soluble sugar and H_2_O_2_ after root induction. Auxin, soluble sugars and H_2_O_2_ levels were higher in rejuvenated shoots than in mature shoots, whereas the opposite was true for ABA and GAs. Notably, indole-3-acetic acid (IAA) and GA3 presented higher contents with more obvious differences in *T. hybrid* ‘Zhongshanshan 302’ rejuvenated shoots vs. mature shoots compared with other kinds of auxin and GAs. The evident improvement in the rooting ability of rejuvenated shoots after grafting likely resulted from the differential regulation of plant hormones, carbohydrates and redox signaling. In addition to the physiological basis of improved rooting ability by grafting, this study provided a theoretical basis for the optimization of subsequent propagation techniques in *T. hybrid* ‘Zhongshanshan’ and potentially other *Taxodium* spp.

## 1. Introduction

*Taxodium hybrid* ‘Zhongshanshan’ is an interspecies hybrid clone generated from *T. mucronatum*, *T. distichum* and *T. ascendens* [1]. As it has strong resistance to waterlogging, salt and other stresses, and high use value, *T. hybrid* ‘Zhongshanshan’ has been widely used for ecological restoration, saline-alkali land greening, coastline protection and landscaping in southeastern China [2,3,4,5]. The use of cuttings is the main method currently used to propagate *T. hybrid* ‘Zhongshanshan’ to meet ecological and economic needs [6,7]. However, the older the physiological age of the mother tree, the weaker the capacity of cuttings forming adventitious roots, and the more difficult for propagation by cuttings [6]. As a result, it is important to improve the adventitious root formation (ARF, herein comprising root induction, initiation, and emergence stages) capacities of mature trees.

Trees that reach the adult or reproductive stage can regain their juvenile characteristics through a variety of processes [8]. Juvenile and rejuvenated trees have many advantages in growth vitality, including a significantly improved ARF [9]. Rejuvenation can be achieved by artificial methods, such as grafting, pruning, chemical treatments, etc. [10,11,12] The rooting ability of micro-cuttings in *Sequoia sempervirens* and *Castanea sativa* can be restored by serial micro-grafting [13,14]. Mature walnut trees could be rejuvenated by horizontal grafting and burying, which improved the rooting capacity [9].

ARF in stem cuttings is controlled by many factors such as genetic information and environment, in which phytohormones play important roles [15]. For example, exogenous auxin has been used to increase the ARF of cuttings in several plant species [16]. In addition, ARF is induced by the high level of auxin and low level of cytokinin [17]. Other hormones, such as abscisic acid (ABA) and gibberellins (GAs), have also been reported as having a phase-dependent effect in ARF [18]. Auxin could also increase the production of hydrogen peroxide, which is essential for increasing the ARF of cuttings [19]. In addition, it was found in previous studies that the trend of ARF response was consistent with the trend of soluble sugar content [17]. Juvenile and mature trees differ in terms of phenotype, structural organization, physiology and gene expression profiles [9]. Plant hormones, redox signaling and carbohydrates play an important regulatory role in the processes of rejuvenation and inducing ARF. Rooting abilities of successive generations of *Buxus sinica* cuttings are associated with changes in the levels of indole-3-acetic acid (IAA), abscisic acid (ABA) and gibberellins [20]. Redox signaling, which interacts with phytohormones pathways, exerts effects on plant development [21]. Several changes in ROS generation and scavenging are age related in some organisms [22]. It has been reported that H_2_O_2_, the most abundant plant ROS, was involved in ARF in different plant species [23]. It was also found that the enhanced rooting efficiency was probably due to high sugar content, which might be beneficial for adventitious root primordium development in rejuvenated shoot cuttings [24,25].

We found an improved ARF in rejuvenated *T. hybrid* ‘Zhongshanshan’ shoots from mature trees after grafting. However, our understanding of how the factors altered during the rejuvenation process improves ARF is quite limited. As an initial approach to address this problem, we investigated herein the temporal changes in the levels of endogenous hormones, H_2_O_2_ and soluble sugars underlying rejuvenation and the consequent improved ARF in *T. hybrid* ‘Zhongshanshan’.

## 2. Results

### 2.1. Rooting of Mature and Rejuvenated Soft Shoots

**Figure 1:**
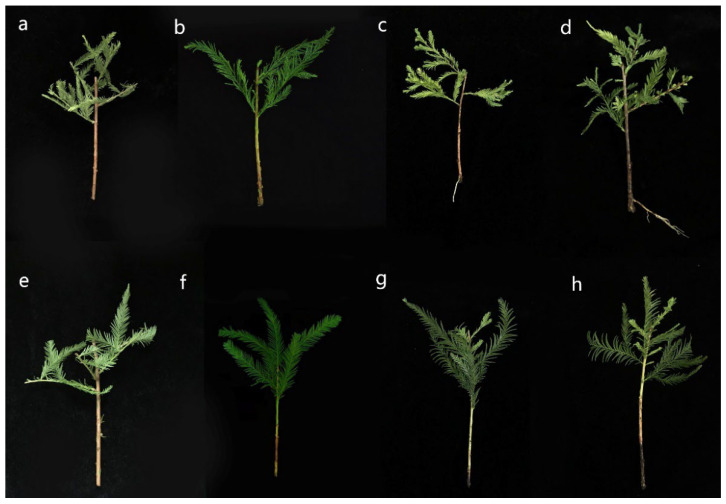
Adventitious root formation in mature and rejuvenated soft shoots of *T. hybrid* ‘Zhongshanshan 302’ at different developmental times. (**a**–**d**) rejuvenated soft shoots at 1st, 21st, 42nd and 56th days, (**e**–**h**) mature soft shoots at 1st, 21st, 42nd and 56th days.

We found that the grafting of young shoots from mature *T. hybrid* ‘Zhongshanshan 302’ trees promoted their rejuvenation and improved ARF. Under the same condition of rooting induction, rooting significantly differed between non-rejuvenated and rejuvenated shoots of *T. hybrid* ‘Zhongshanshan 302’ (Table 1 and Figure 1). In rejuvenated soft shoots, calli were initial induced after 21 days of cutting (induction stage), and the calli formation rate was 61% (Figure 1b). Adventitious roots were detected on the 42nd day (initiation stage), and by day 56 (expression stage), more than half of the adventitious roots broke through the epidermis, with a rooting rate of 51.1% (Figure 1c). In contrast, callus was not evident in the basal regions of 65.1% non-rejuvenated shoots after 21 days of inducing. The basal regions of 69.8% of the non-rejuvenated shoots were blackened and rotted at the 56th day after rooting induction (Figure 1f), and exhibited only a 5% rooting rate.

### 2.2. Quantitative Analyses of Endohormone Levels

**Figure 2:**
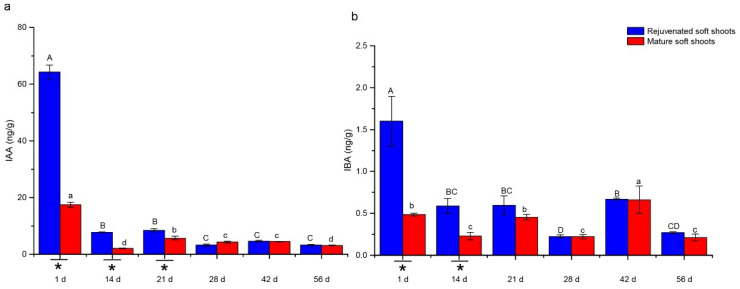
Changes of IAA (**a**) and IBA levels (**b**) in rejuvenated and mature soft shoots of *T. hybrid* ‘Zhongshanshan 302’ during ARF. Different capital letters represent significant differences at *p* ≤ 0.05 among rejuvenated soft shoots at different timepoints, different lowercase letters represent significant differences at *p* ≤ 0.05 among mature soft shoots at different timepoints, and an asterisk represents significant differences at *p* ≤ 0.05 between rejuvenated and mature soft shoots.

**Figure 3:**
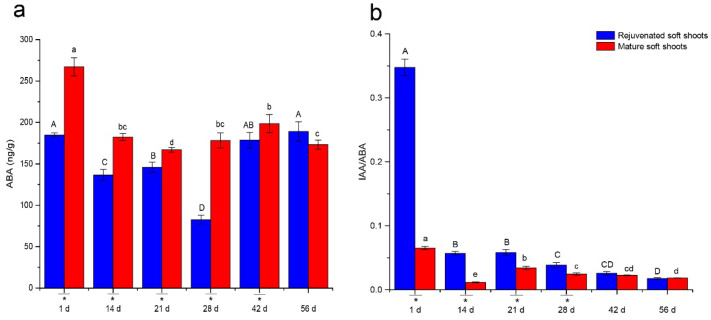
Changes of ABA levels (**a**) and the IAA/ABA ratio (**b**) in rejuvenated and mature soft shoots of *T. hybrid* ‘Zhongshanshan 302’ during ARF. Different capital letters represent significant differences at *p* ≤ 0.05 among rejuvenated soft shoots at different timepoints, different lowercase letters represent significant differences at *p* ≤ 0.05 among mature soft shoots at different timepoints, and an asterisk represents significant differences at *p* ≤ 0.05 between rejuvenated and mature soft shoots.

**Figure 4:**
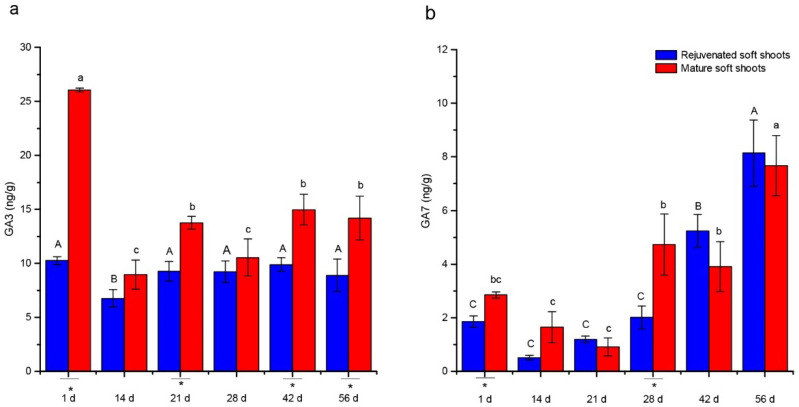
Changes of GA3 (**a**) and GA7 (**b**) levels during ARF in rejuvenated and mature soft shoots of *T. hybrid* ‘Zhongshanshan 302’. Different capital letters represent significant differences at *p* ≤ 0.05 among rejuvenated soft shoots at different timepoints, different lowercase letters represent significant differences at *p* ≤ 0.05 among mature soft shoots at different timepoints, and an asterisk represents significant differences at *p* ≤ 0.05 between rejuvenated and mature soft shoots.

**Figure 5:**
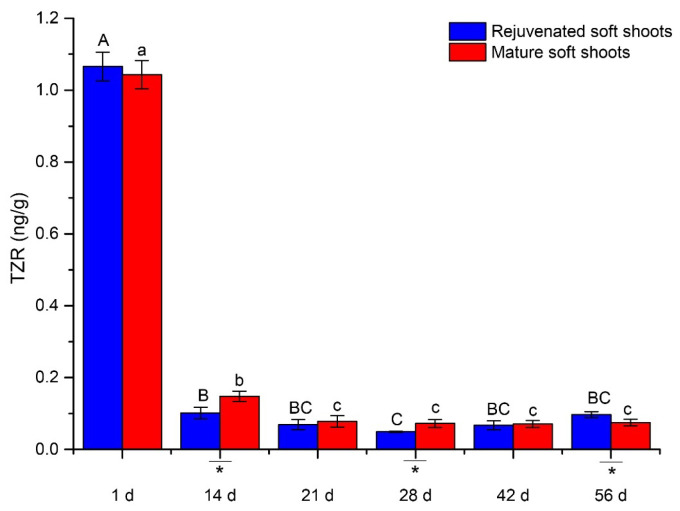
Changes of TZR levels during ARF in rejuvenated and mature soft shoots of *T. hybrid* ‘Zhongshanshan 302’. Different capital letters represent significant differences at *p* ≤ 0.05 among rejuvenated soft shoots at different timepoints, different lowercase letters represent significant differences at *p* ≤ 0.05 among mature soft shoots at different timepoints, and an asterisk represents significant differences at *p* ≤ 0.05 between rejuvenated and mature soft shoots.The IAA contents in rejuvenated shoots were found to be 3.69 times higher than in mature soft shoots on the initial day of rooting (Figure 2a), after which the IAA levels decreased and remained significantly different until the 21st day. In the expression stage (42nd to 56th days), IAA contents were between 3.15–4.57 ng·g^−1^ and did not significantly differ between shoot type. The IBA contents of shoots were comparatively lower and remained in a low range (under 2 ng·g^−1^) during the rooting process (Figure 2b), and followed a decreasing trend similar to that of IAA, with significant differences at 1 and 14 days.

The levels of endohormones in rejuvenated and mature soft shoots were measured by LC-MS/MS and are shown in Figure 2, Figure 3, Figure 4 and Figure 5. The levels of all endohormones in the rejuvenated soft shoots differed significantly from that of mature soft shoots.

During adventitious root formation, ABA contents decreased and then increased, with valleys at the 21st (mature soft shoots) and 28th days (rejuvenated soft shoots) (Figure 3). However, with the exception of day 56, endogenous ABA contents in rejuvenated soft shoots were significantly lower than those in mature soft shoots. This was especially true at day 1, when the ABA content was 82.4 ng/g lower than that in mature soft shoots. The ratio of IAA and ABA was also calculated and showed a continuous downward trend. Nevertheless, the IAA/ABA ratio remained significantly higher in rejuvenated shoots than in mature shoots until 42 days.

Endogenous GA3 and GA7 contents showed changes comparable to those of ABA during ARF (Figure 4). The lowest value of GA3 was found on the 14th day, after which GA3 contents were maintained relatively higher or stable. The level of GA7 content was relatively higher in the expression stage compared with other stages. Rejuvenation also significantly affected the levels of endogenous GA contents (Figure 4). Endogenous GA3 contents in rejuvenated shoots were mostly significantly lower than mature soft shoots during ARF, whereas GA7 content showed less significant differences between rejuvenated and mature soft shoots.

Three endogenous cytokinins, trans-zeatin riboside (tZR), zeatin and IP, were detected in this study, but only tZR was present in sufficient quantities for monitoring during ARF (Figure 5). In the process of ARF, tZR levels decreased significantly after root induction in both rejuvenated and mature cuttings. However, at day 14, 28 and 56, the decline was more pronounced in the rejuvenated shoots.

### 2.3. Analyses of H_2_O_2_ and Sugar

**Figure 6:**
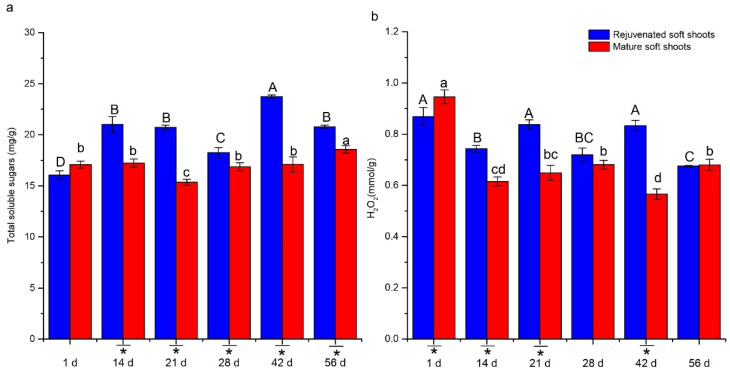
Changes of soluble sugars (**a**) and H_2_O_2_ (**b**) contents in rejuvenated and mature soft shoots of *T. hybrid* ‘Zhongshanshan 302’ during AFR. Different capital letters represent significant differences at *p* ≤ 0.05 among rejuvenated soft shoots at different timepoints, different lowercase letters represent significant differences at *p* ≤ 0.05 among mature soft shoots at different timepoints, and an asterisk represents significant differences at *p* ≤ 0.05 between rejuvenated and mature soft shoots.

Rejuvenated shoots also displayed significantly altered contents of soluble sugars and H_2_O_2_ relative to mature shoots (Figure 6). The content of total soluble sugars in rejuvenated soft shoots was higher than mature soft shoots during the whole rooting process except on the initial day. It followed a bimodal profile, with peaks at 14 and 42 days. In contrast, total soluble sugars in mature soft shoots decreased and then increased, with a valley at the 21st day. Significant differences were also observed in H_2_O_2_ content between rejuvenated and mature soft shoots. Compared with mature soft shoots, rejuvenated soft shoots maintained a relatively higher level during the first 21 days.

## 3. Discussion

The transition of trees from juvenile to adult usually reduces and inhibits ARF in various degrees. For example, the rooting rate of 3-year old cuttings (88%) of *Rhamnus caroliniana* Walt. was significantly higher than that of 36-year old cuttings (17%) [26]. Certain rejuvenation treatments, such as grafting onto juvenile rootstock, the application of growth regulators and others, may improve the ARF from shoots formed on mature trees [8], including walnut [9] and chestnut [13]. *T. hybrid* ‘Zhongshanshan 302’ is an elite variety of *Taxodium* that was selected in 1979, derived from F1 generations resulting from a cross between *T. distichum* and *T. mucronatum*. It grows fast, has tall and straight trunks, and tolerates waterlogging, submerging, saline, and storms [27]. However, the rooting rate of *T. hybrid* ‘Zhongshanshan 302’ was significantly lower than that of *T. hybrid* ‘Zhongshanshan 118′ that was selected in 1993 and *T. hybrid* ‘Zhongshanshan 405′ that was selected in 2004 [28]. In addition, as physiological age increases, rooting rates of *T. hybrid* ‘Zhongshanshan 302’ cuttings tend to decrease, similar to chestnuts, thereby greatly limiting its popularization and application [29]. In this study, we have shown that grafting effectively improved the rate of rooting of shoots from mature *T. hybrid* ‘Zhongshanshan 302’ (Table 1 and Figure 1). This method has also been successfully used to rejuvenate *Pinus massoniana*, with rooting rates greater than 83.1% [30].

We also found that rejuvenation induced changes in the physiological properties of mature soft shoots. Plant hormones play important regulatory roles in the process of plant rejuvenation. The morphological change of adventitious root development is accompanied by the regulation of endogenous hormones. The differences in rooting ability of successive generations of *Buxus sinica* var. parvifoli cuttings was associated with changes in the levels of endohormones, including auxin, abscisic acid and gibberellin [20]. A positive relationship was found between IAA level and the rooting response in almond [31]. Endogenous ABA may be closely associated with the inhibition of root formation and an enhanced rooting ability of explants of *Sequoia* is closely related to a high IAA/ABA ratio [32]. During the rejuvenation of *Sequoia sempervirens* shoots, the recovery of rooting ability was reported to be regulated by both auxin and ABA [33]. In the present study, auxin levels decreased during the ARF process. However, the level of IAA was higher than that of IBA and the levels of IAA and IBA in the rejuvenated shoots were significantly higher than those in the mature shoots, suggesting that the improved rate of ARF in rejuvenated shoots could be related to the regulation of auxin in *T. hybrid* ‘Zhongshanshan 302’. On the contrary, ABA contents in rejuvenated shoots were lower than in mature shoots during most of the ARF stage. ABA can interact with IAA and is required for ARF, but high levels will inhibit this process [34,35,36]. Therefore, it is possible that the rejuvenation process reduced the concentration of ABA in *T. hybrid* ‘Zhongshanshan 302’. In addition, the endogenous IAA/ABA ratio was found to be a reliable marker for rejuvenation [30]. This study is in agreement with this observation, because the IAA/ABA ratio was maintained significantly higher in rejuvenated shoots than mature shoots until the expression stage of ARF at 42 days.

Cytokinins also regulate adventitious root formation in a stage-specific manner and an early decrease in cytokinins is thought to contribute to the induction of ARF when combined with high auxin levels [37,38]. The contents of endogenous cytokinins were proposed as a marker for rejuvenation in tissue culture plantlets in *Hevea brasiliensis* [39]. In this study, the rejuvenated and mature cuttings both displayed a decrease in tZR levels, but this decrease was more pronounced at 14 days in the rejuvenated shoots, which was in agreement with the general observation that cytokinin depletion participates in the induction of adventitious roots in cuttings [38]. GAs have been shown to significantly inhibit ARF and development [40]. External application of GAs has been shown to inhibit rooting, possibly by inhibiting auxin polar transport [41]. Furthermore, the species that are recalcitrant to rooting were found to have high endogenous GA content, indicating that high endogenous GA content may be a limiting factor [42]. Conversely, some studies have shown that endogenous GA1 was necessary for AR formation, which seemed to be related to the functional differences of different types of GAs [43]. Our findings indicated that the GA3 level was lower in the rejuvenated soft shoots than in mature soft shoots, but GA7 behaved differently. Therefore, the roles and relationships between different gibberellins appeared to be both complex and context-specific, and warrant further investigation.

H_2_O_2_ and soluble sugars may also play regulatory roles in adventitious root development in *T. hybrid* ‘Zhongshanshan 302’, because the levels of these two factors were also affected by the rejuvenation treatment. Redox homeostasis was reported to play a role in the induction, differentiation and/or formation of adventitious roots, and the accumulation of H_2_O_2_ in the differentiation zone acts as a signal molecule in the formation of adventitious root [32]. In addition, hydrogen peroxide may cooperate with auxin in the improvement of ARF [44]. We observed that rejuvenated soft shoots maintained relatively higher H_2_O_2_ and IAA levels during the initiation stage of ARF compared with mature soft shoots. Furthermore, auxin and H_2_O_2_ can promote an increase in soluble sugars during root development [16]. The enhanced rooting efficiency of etiolated coppice-shoot cuttings, which might be due to an enhanced adventitious root primordium development, was suggested to be due to higher sugar availability [24,45]. Higher levels of soluble sugar were observed in rejuvenated shoots in this study. The higher sugar availability may enhance energy sources and the signaling interaction with hormonal and other pathways to positively promote ARF and development in *T. hybrid* ‘Zhongshanshan 302’.

## 4. Materials and Methods

### 4.1. Plant Material and Root Induction

The mature woody plant material was ca. 30-year-old *T. hybrid* ‘Zhongshanshan302′, exhibiting healthy growth and located in Jingjiang, Jiangsu, China. Rejuvenated soft shoots were obtained as follows: briefly, 1–2-year-old well-grown *T. distichum* (L.) Rich seedlings were selected as rootstock; 1–2-year-old branches of the mature *T. hybrid* ‘Zhongshanshan 302’ were cut as scion strips before budding in spring and grafted onto the rootstock by cut-grafting; and newly sprouted branches from the scion were cut again as scion strips and grafted in next year. After grafting four times, newly sprouted branches were trimmed as rejuvenated shoots for cuttings. Mature soft shoots were collected from the 30-year-old stock tree for cuttings for comparison. Each cutting was cut into a length of 15 cm and one-half of the leaves were removed. The cuttings were planted into the seedbed (containing moistened perlite: peat soil, 1:1, which had been exposed to the sun for sterilization) in a nursery with a sunscreen and spray device to maintain 80% humidity and a temperature of ca. 30 °C without using other chemical substances such as pesticides. The incidence of rooting was monitored at the 1st, 7th, 14th, 21st, 28th, 35th, 42nd, 49th and 56th days after rooting induction. The calli formation rate was calculated as the ratio of the number of cuttings with callus at the base and the number of all cuttings. The scale is referred to in Figure 2b. The rooting rate was calculated as the ratio of the number of cuttings with adventitious roots at the base and number of all cuttings. The scale is referred to in Figure 2c. A minimum of 100 soft shoots were sampled at each time point to measure the calli formation rate and the rooting rates. In addition, the basal stem tissue (0.5 cm) and the root tissue of the cuttings were collected at specific time points of adventitious root development (Figure 1), immediately frozen in liquid nitrogen, and stored at −80 °C until analysis. There were three biological replicates per sample for later quantification of endogenous hormone levels, H_2_O_2_ and soluble sugar.

### 4.2. Quantification of Endogenous Hormone Levels

A quantitative analysis of plant endogenous hormones was performed according to previously reported methods [46]. One g of tissues was snap-frozen and ground in liquid nitrogen, followed by 2 x extraction with isopropyl alcohol/hydrochloric acid extraction buffer and dichloromethane. The extract was then concentrated at 13,000× *g* for 5 min at 4 °C. The lower organic phase was taken to be dried under nitrogen in the dark and then dissolved in methanol containing 0.1% formic acid, filtered through a 0.22 µm membrane combined with a poroshell 120 SB-C18 Cartridge (Agilent Technologies, Palo Alto, CA, USA) after adding 8 μL internal standard (1 μg/mL). The eluate was analyzed using an HPLC-MS/MS system consisting of an Agilent 1290 HPLC system coupled to an SCIEX-6500 Qtrap mass spectrometer (Applied Biosystems/MDS Sciex, Concord, ON, Canada). The quantification of identified endogenous hormones was calculated from their peak areas relative to that of the standard solution, of which the solvent was methanol containing 0.1% formic acid (0.1, 0.2, 0.5, 2, 5, 20, 50, or 200 ng/mL of IAA, IBA, ABA, TZR, Zeatin, IP, IPA, GA1, GA3, GA4, or GA7, with internal standard solution).

### 4.3. Analyses of H_2_O_2_ and Soluble Sugar

The assay of H_2_O_2_ was carried out according to the method described by Liu et al. [47] using kits that were provided by the Nanjing Jiancheng Bioengineering Institute (Nanjing, China). One hundred mg of tissues was homogenized in ice-cold potassium phosphate buffer (50 mM, pH 7.8) and 1 mL of the supernatant (1 mL) was mixed with 0.1 mL of molybdic acid before the content of H_2_O_2_ was determined spectrophotometrically at 405 nm.

The concentration of soluble sugar was determined using the anthrone-sulfuric acid method as described previously [16]. Two hundred mg of fine powder of bark samples was extracted in double distilled water in a boiling water bath for 30 min and then centrifuged at 6000× *g* for 10 min at room temperature. After the collection of the first supernatant, the process was repeated and the supernatants combined. The supernatant was then gently mixed with anthrone-ethyl acetate reagent and concentrated sulfuric acid solution and heated in a boiling water bath for 1 min. After cooling, the concentration of soluble sugar was measured spectrophotometrically at 630 nm. The amount of soluble sugar was calculated based on a standard linear curve.

### 4.4. Data Analysis

All data were presented as means ± standard deviations (SD). Statistical analysis among timepoints was performed by one-way ANOVA followed by the LSD test after analyzing the normal distribution with a histogram of the distribution plot with the normal distribution curve using the statistical program SPSS 21.0 for Windows (www.spss.com, accessed on 1 August 2022). Differences between rejuvenated and mature soft shoots were evaluated using the t-test for independent samples after analyzing the normal distribution as well. Values were considered statistically different when *p* < 0.05. All figures were drawn using OriginPro 9.1 software.

## 5. Conclusions

The evident improvement in the rooting ability observed after the rejuvenation of shoots by grafting likely resulted from the differential regulation of plant hormones, carbohydrates and redox signaling, because the improved ARF was associated with elevated auxin, soluble sugars and H_2_O_2_, and depressed ABA and GAs. In particular, IAA and GA3 may play key roles in *T. hybrid* ‘Zhongshanshan’ rejuvenation compared with other kinds of auxin and GAs. The roles and relationships between different hormone species warrant further investigation. This study not only analyzed the physiological changes associated with the improvement of shoot rooting after rejuvenation, but also provided a theoretical physiological basis for the optimization of propagation techniques in *T. hybrid* ‘Zhongshanshan’. Obviously, other underlying molecular mechanisms, including the regulation analysis of miRNA and DNA methylation, need to be studied further.

## Figures and Tables

**Table 1:** Rooting and callus formation statistics of mature and rejuvenated soft shoots of *T. hybrid* ‘Zhongshanshan 302’.

		Treatments	Calli Formation Rate (%)	Rooting Rate (%)
Induction stage	1st day	Rejuvenated soft shoots	0	0
		Mature soft shoots	0	0
	7th day	Rejuvenated soft shoots	0	0
		Mature soft shoots	0	0
	14th day	Rejuvenated soft shoots	0	0
		Mature soft shoots	0	0
	21st day	Rejuvenated soft shoots	61.0	0
		Mature soft shoots	34.9	0
Initiation stage	28th day	Rejuvenated soft shoots	67.5	0
		Mature soft shoots	33.3	0
	35th day	Rejuvenated soft shoots	76.4	0
		Mature soft shoots	38.1	0
Expression stage	42nd day	Rejuvenated soft shoots	69.6	4
		Mature soft shoots	31.4	0
	49th day	Rejuvenated soft shoots	69.3	16.8
		Mature soft shoots	42.6	0
	56th day	Rejuvenated soft shoots	75.6	51.1
		Mature soft shoots	25.2	5.0

## Data Availability

Data is contained within the article.

## Abbreviations

HPLC	High performance liquid chromatography
iP	N^6^-(Δ^2^-isopentenyl)-adenine
iPA	N^6^-(Δ^2^-isopentenyl)-adenosine
tZR	Trans-zeatin riboside
ABA	Abscisic acid
GAs	Gibberellins
IAA	Indole-3-acetic acid
IBA	Indole-3-butyric acid
ROS	Reactive oxygen species
ARF	Adventitious root formation
